# Supplementary material for: Integrated Secondary Metabolomic and Antioxidant Ability Analysis Reveals the Accumulation Patterns of Metabolites in Momordica charantia L. of Different Cultivars
Source: Int J Mol Sci. 2023 Sep 24;24(19):14495. doi: 10.3390/ijms241914495 (PMC10572697; doi:10.3390/ijms241914495)
Supplement: Supplementary file 1 [file ijms-24-14495-s001.zip › Figure S1.pdf]

A

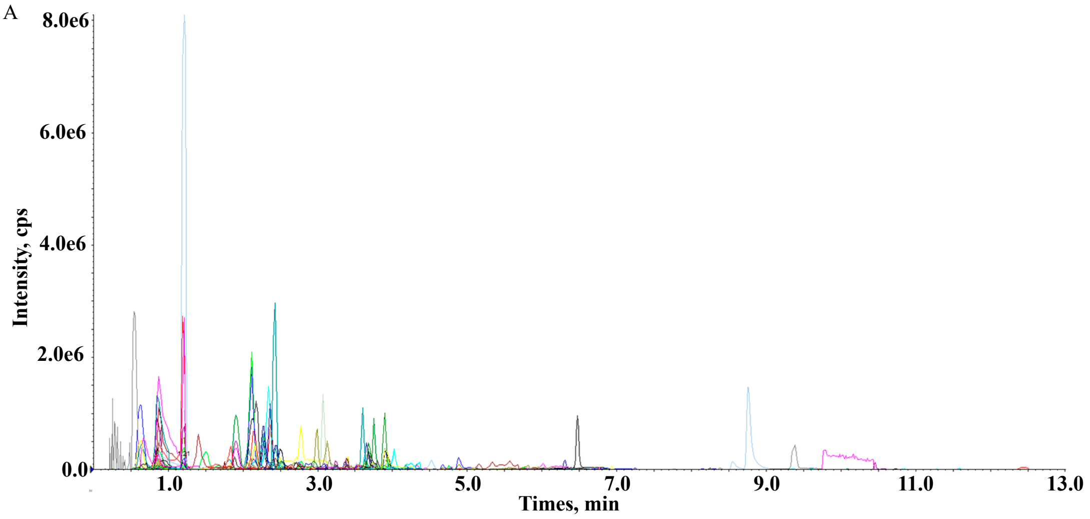

8.0e6
6.0e6
4.0e6
2.0e6
0.0
Intensity, cps
1.0
3.0
5.0
7.0
9.0
11.0
13.0
Times, min

B

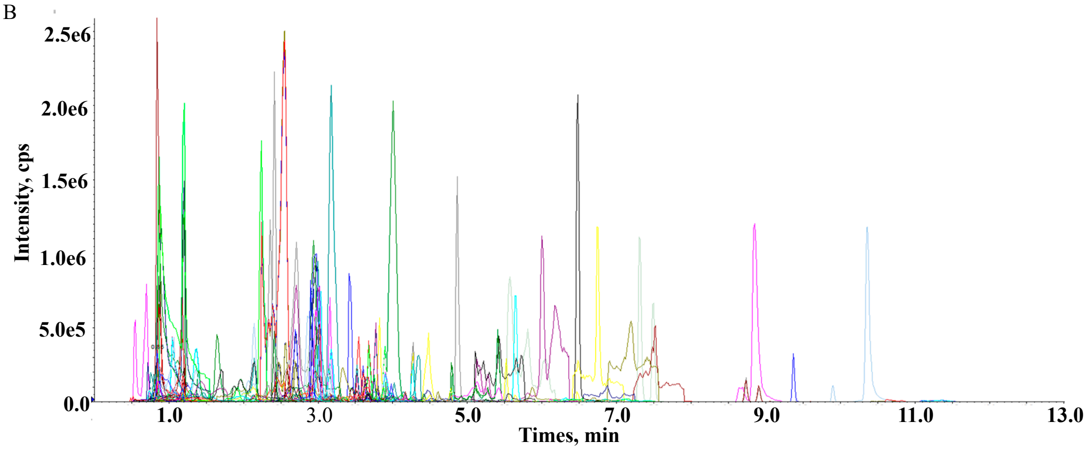

2.5e6
2.0e6
1.5e6
1.0e6
5.0e5
0.0
Intensity, cps
1.0
3.0
5.0
7.0
9.0
11.0
13.0
Times, min

C

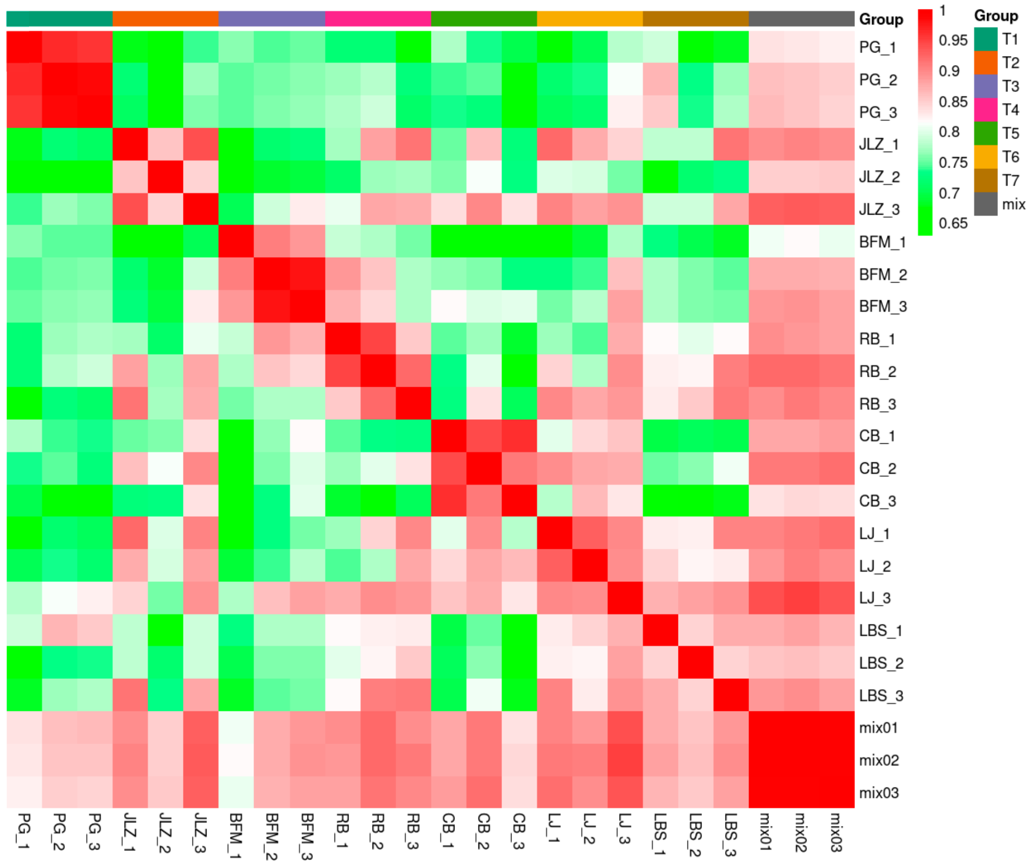

Group
PG_1
PG_2
PG_3
JLZ_1
JLZ_2
JLZ_3
BFM_1
BFM_2
BFM_3
RB_1
RB_2
RB_3
CB_1
CB_2
CB_3
LJ_1
LJ_2
LJ_3
LBS_1
LBS_2
LBS_3
mix01
mix02
mix03
1
0.95
0.9
0.85
0.8
0.75
0.7
0.65
Group
T1
T2
T3
T4
T5
T6
T7
mix

Figure S1. Overview of the metabolomics analysis. (A, B) The total ion current (TIC) of the QC samples in positive ion mode and negative ion mode. (C) Pearson's correlation coefficient analysis of PG, JLZ, BFM, RB, CB, LJ, LBS and Mix quality control sample.
